# Case Report: Treating depression and alexithymia in a 15-year-old girl: an emotion-focused therapy case study

**DOI:** 10.3389/fpsyt.2025.1715173

**Published:** 2026-01-12

**Authors:** Feifei Xu, Hang Zhang

**Affiliations:** 1 School of Humanities and International Education Exchange, Anhui University of Chinese Medicine, Hefei, China; 2 School of Psychology, Zhejiang Normal University, Jinhua, China

**Keywords:** alexithymia, case study, depression, emotion-focused therapy, psychotherapy

## Abstract

**Objective:**

This single-case study aimed to examine the effects of emotion-focused therapy (EFT) on the psychological status of a 15-year-old female high school student diagnosed with depression and alexithymia.

**Methods:**

The study employed a single-case design comprising 18 sessions of EFT. Psychological assessment was conducted using the Symptom Checklist-90-Revised (SCL-90-R), Self-Rating Depression Scale (SDS), Self-Rating Anxiety Scale (SAS), and the Toronto Alexithymia Scale (TAS-20). Data obtained throughout the counseling process were systematically organized and analyzed.

**Results:**

Pre-intervention scores were as follows: SCL-90-R total score = 190, SDS = 72, SAS = 60, and TAS-20 = 60. Post-intervention assessments indicated notable improvements across all measures: SCL-90-R total score = 140, SDS = 45, SAS = 38, and TAS-20 = 45.

**Conclusion:**

The results suggest that emotion-focused therapy may effectively reduce emotional avoidance and improve the acceptance and comprehension of negative emotional experiences by facilitating the progressive processing of core emotions.

## Introduction

Depression is a globally prevalent psychiatric disorder, with major symptoms including low mood, irritability, decreased concentration, loss of interest in previously preferred activities, and suicidal thoughts (1) Previous evidence shows that depression not only affects physical and mental health but also indirectly reduces research and work productivity, suggesting that depression has become an increasingly important public health issue (2). Therefore, it is necessary to pay attention to the diagnosis and treatment of depression to reduce the personal and social burden.

Alexithymia is a personality construct involving difficulty in identifying and describing emotions, as well as an externally oriented cognitive style.

These individuals often resort to negative coping strategies, such as suppression, denial, and distortion, in managing their emotions. Positive coping strategies act as a buffer in alleviating emotional distress induced by stress, whereas negative coping strategies may aggravate emotional symptoms under stress. Furthermore, prolonged neglect of negative emotions and a lack of effective strategies for managing them result in not only in deficient emotional regulation skills but also in the gradual development of negative beliefs about oneself, the environment, and the future. Individuals may perceive themselves as worthless and incapable, the environment as overly stressful, and the future as hopeless (3, 4). These perceptions further exacerbate feelings of loneliness, vulnerability, and inefficacy, leading to unhealthy behaviors such as social withdrawal. Such withdrawal behaviors, in turn, hinder the accumulation of new positive experiences related to emotional experience and regulation, reducing the likelihood of modifying the original maladaptive processing mechanisms. Alexithymia involves at least three core aspects: Identification Difficulty (DIF), description difficulty identifying feelings (DIF), difficulty describing feelings (DDF), and externally oriented thinking (EOT) (5). While considering the importance of depression, the dimensions related to difficulties in emotion recognition and emotional communication have been shown to be positively correlated with depression and anxiety (6). Researchers have used the alexithymia and depression scales to measure the symptoms in college students, and analyzed the results. The significant factor loadings of each scale item showed minimal overlap, and the factors were closely related to their respective constructs. These results were replicated and cross-validated in a psychiatric outpatient sample. These findings support the view that alexithymia is a distinct and distinct construct, separate from depression (7). Therefore, individuals with alexithymia also require further attention, and there is a need to identify treatment approaches that focus on individual emotional processing to improve the efficiency of treatment efficacy.

At the same time, depression is the most prevalent psychological disorder among adolescents, ranking second among the 25 leading causes of disability in this group (8). Adolescent depression is a widespread mental illness with significant burdens and costs to society, education, interpersonal relationships, the economy, and future developmental outcomes that are compromised. Many vulnerability factors increase the risk of depression in adolescents, especially in the face of stressful life events. Therefore, adolescents with elevated risk factors or high levels of stress should be appropriately assessed and promptly treated, and effective, experience-based treatments should be used to help those with clinical depression (9).

Medication, including the use of antidepressants such as tricyclic antidepressants (TCAs) and serotonin-selective reuptake inhibitors (SSRIs), and psychotherapy are the two main types of treatment available to adolescents. However, medication is associated with side effects (e.g., dizziness, orthostatic hypotension, tremors, and dry mouth) (10) and potential risks (11). In patients who experience treatment-related side effects, symptom improvement may be delayed, and adherence to treatment can be compromised (12). In terms of psychotherapy, a meta-analysis found that after treatment, psychotherapy was significantly more effective than other treatments (e.g., placebo and conventional therapy) and helped depressed adolescents in the short term. None of these studies reported adverse effects or cost-effectiveness outcomes (13).

Emotional-focused therapy (EFT) has unique advantages for alexithymia, mainly reflected in the following aspects. First, EFT emphasizes emotional awareness and acceptance. EFT guides individuals to focus on their physical and emotional experiences, helping them gradually identify and name vague or difficult-to-express emotions, which is a key entry point for improving their emotional cognition. Second, EFT emphasizes a safe space for emotional expression. EFT provides a non-judgmental environment for individuals with expressive disorders, encouraging them to express their emotions through speech, writing, or behavior, and alleviating social anxiety or isolation caused by difficulties in emotional expression. Third, EFT promotes the connection between emotions and needs. EFT guides individuals to explore unmet needs behind emotions (such as a sense of security and belonging), helping individuals with affective disorders understand the functional meaning of emotions, rather than remaining at a superficial emotional level. Finally, EFT emphasizes therapist empathy and guidance. EFT therapists use empathic responses and emotional markers (e.g., “you seem to feel ignored”) to help individuals with affective disorders validate the legitimacy of their emotional experiences and reduce their denial or suppression of emotions (14). Emotion-focused therapy aims to assist patients in emotionally processing their underlying maladaptive emotions, transforming their emotional suffering and distress, thereby enabling adaptive emotional responses and reactions to situations. Emotional processing has been recognized as a vital change mechanism of change and has been shown to predict outcomes across various therapeutic modalities (15). In EFT, emotional processing involves approaching, accepting, tolerating, expressing, regulating, understanding, utilizing, and transforming maladaptive emotions with emerging adaptive emotions. Despite extensive research on the psychological treatment of adolescent depression, there is a lack of reports specifically examining depressed patients with alexithymia (16). The aim of this study is to explore the effects of emotion-focused therapy on the psychological state of adolescents with depression and alexithymia through a case study, and to provide support for clinicians using emotion-focused therapy in the treatment of adolescent depression patients.

## Research methodology

### Patient

The patient is a 15-year-old girl in her third year of junior high school. She is the second child in the family, with a sister who is 3 years older. After her parents divorced when she was 8 years old, she was raised by her father, with minimal interaction between her parents. The patient has demonstrated excellent academic performance and has served as a class committee member and Student Union member, fulfilling her duties effectively. She was diagnosed with moderate depression in the seventh grade but refused medication. The patient has not engaged in any behaviors that harm herself or others. Information disclosure related to this study was conducted with the consent of the patient and her guardians, who signed informed consent forms and personal information disclosure forms, agreeing to the use of the information.

### Chief complaint and personal statement

#### Chief complaint

The patient reports persistent low mood and a lack of close friendships. She describes a significant difficulty in expressing and identifying her own feelings. For instance, even when experiencing physical symptoms of fear, such as trembling, she remains unclear about what she is feeling. Furthermore, she is unable to accurately distinguish between emotions such as sadness, fear, and anger, which causes her considerable distress. As holidays approach, she expresses reluctance to return home and face her family.

#### Personal statement

For the past 2 years in junior high school, I have felt a profound lack of intimate friendships, spending each day in solitude. Although I desire connection, I find myself with nothing to say to my roommates and classmates, and over time, I have lost the impulse to talk, preferring to be alone. This has fostered a persistent sense of disengagement, as if I am not truly part of anyone’s life. My mood has been consistently low. I feel numb every day, unable to feel happiness, though not exactly sad. Nothing interests me—not hobbies, nor classes. I struggle to listen and often consider skipping school. When my depression is severe, it manifests as headaches. I feel I have no purpose, no interests, and no direction for the future. I describe myself as a “hollow tree,” consumed by a pain that has no solution, and I hate myself for being in this state. I have had thoughts of death in the past, and they persist. To me, death feels like something tragic yet romantic. With the summer vacation approaching, I feel no desire to go home. I have nothing to talk about with my family, and being at home sometimes makes me uncomfortable and fills me with a sense of guilt toward them.

### First impressions of consulting

At the first meeting, the patient was observed to have a normal appearance. Her speech was slow and soft, accompanied by rigid body language, and she showed minimal emotional reactivity. Her narrative was characterized by a focus on external events rather than internal emotional states, a trait associated with alexithymia. A key clinical feature was her difficulty in discriminating between emotions and bodily sensations, leading to the somatization of distress into symptoms such as headaches and chest tightness.

### Function

Mental state: The patient is conscious and oriented. A depressed mood was observed. Her speech and behavior were congruent with her affect. No bizarre or dysfunctional behaviors were noted, indicating a relatively stable personality.

Physical state:

Nearly 1 year of poor sleep; appetite is not strong.

Social function: She exhibits withdrawn behavior, demonstrating a reluctance to communicate with others. Interpersonal relationships with classmates are distant, and there is a notable lack of interaction with both peers and teachers. Furthermore, she has difficulty maintaining concentration in.

### Diagnosis

Depressive disorder was diagnosed according to the Diagnostic and Statistical Manual of Mental Disorders (Fifth Edition, Text Revision) (DSM-5-TR).

### Measures

#### Symptom Checklist-90-Revised (SCL-90-R)

The SCL-90-R was developed by Derogatis LR and is currently one of the most widely used screening scales for mental disorders and psychological disorders. The scale consists of 90 items, including 10 factors: somatization, obsessive–compulsive symptoms, interpersonal sensitivity, depression, anxiety, hostility, phobic anxiety, paranoia, psychoticism, and sleep and eating status. The scale adopts a 5-point Likert scoring method. Each item is rated from 1 to 5 (“never” = 1 point, “very mild” = 2 points, “moderate” = 3 points, “heavy” = 4 points, and “severe” = 5 points), with higher total scores indicating poorer mental health status. If the total score is ≥160 points or above, or if the number of positive items (score ≥ 2 points) exceeds 43, or if any factor score exceeds 2 points, the screening is considered positive for psychological abnormalities (17).

#### Self-Rating Anxiety Scale and Self-Rating Depression Scale

Before and after the intervention, the SAS and SDS were used for evaluation. Both scales consist of 20 items, each scored from 1 to 4, with a total score of 80. A score of <53 indicates normal emotional status, while a score ≥53 suggests the presence of anxiety or depressive symptoms in patients. The standard score is calculated by multiplying the original total score multiplied by 1.25 and taking the integer value. This study used standard scores. The Cronbach’s α coefficients for the SAS and SDS were 0.733 and 0.765, respectively (18, 19).

#### Toronto Alexithymia Scale (TAS-20)

This study used the revised Chinese version of the scale. The TAS-20 consists of 20 items and includes three factors: difficulty identifying feelings, difficulty describing feelings, and externally oriented thinking. Difficulty identifying feelings refers to challenges in recognizing one’s own and others’ emotions, such as being unable to accurately identify changes in others’ facial expressions. Difficulty describing feelings refers to obstacles in describing one’s own emotions (20).There are obstacles, such as an inability to express inner thoughts or emotions using accurate vocabulary. Externally oriented thinking refers to individuals paying little or no attention to their inner feelings, lacking imagination, and focusing primarily on external details of daily life. Each item on this scale is scored on a scale of 1-5-point scale, with 1 indicating complete disagreement and 5 indicating complete agreement. Higher scores indicate a more severe degree of emotional expression disorder. The Cronbach’s α coefficient of the scale is 0.81.

This study completed two measurements: the first (T1) was conducted at baseline and prior to the intervention, and the second measurement (T2) was conducted after completion of the intervention completion. No follow-up assessments were performed.

### Treatment process

Using emotion-focused therapy (EFT), the intervention was administered by physicians holding a clinical psychotherapist certification (China). The psychotherapist conducted 18 psychotherapy sessions with the patient. Each session lasted 60 minutes and was held once per week. The interventions were conducted in the psychotherapy room of the hospital’s psychiatric department.

The counseling process was divided into the following three stages. Treatment was delivered by a professionally trained and certified psychotherapist in the hospital and supervised by the department and senior clinicians. Sessions followed the standard EFT case formulation and task structure, and were regularly supervised to ensure adherence to the EFT model. The patient demonstrated good compliance and participated in the entire treatment process. During the treatment period, she did not receive antidepressant medication or other forms of psychotherapy. See **Table 1**.

**Table 1:** Treatment process.

Stage	Session	Time	Techniques	Treatment content
Stage 1	Session 1-6	2024.02-2024.04	empathic concern; Collaborative Focus	Initial Interview and Addressing Resistance and Enhancing Emotional Awareness
Stage 2	Session 7-12	2024.04-2024.06	Empathy arousal, interference elimination, emotional annotation, and interactive pattern recognition	Understanding the Formation and Development of Maladaptive Emotional Processing Mechanisms
Stage 3	Session 13-18	2024.06-2024.08	Double chair/empty chair technology	Establishing Adaptive Emotional Processing Mechanisms

#### Stage 1: Addressing resistance and enhancing emotional awareness

In the initial stages of counseling, the patient resists expressing and exploring her negative emotions. She tends to talk about the positive events in her life, and when it comes to the negative experiences are mentioned, she emphasizes her resilience and minimizes their impact. When asked how she feels internally, she often chooses silence (for example, when the patient talks about a conflict with her family over weekend tutoring—she does not want to attend the tutoring, and experiences physical tension—she describes the situation calmly and expresses little emotion). The therapist responds to this resistance with empathy and provides ample space. As a trusting counseling relationship develops, the patient reveals her belief that emotions, especially negative ones, are not welcome. Through empathy, the counselor affirms the legitimacy of the patient’s emotions, guides her to attend to her bodily sensations, and helps cultivate stronger emotional awareness. By providing feedback on the underlying causes of these feelings, the counselor increases the patient’s acceptance of her emotions.

#### Stage 2: Understanding the formation and development of maladaptive emotional processing mechanisms

The patient becomes aware that she is prone to overreacting to a word or action from a family member or friend, often experiencing intense emotional pain and despair. Through open-ended questioning and emotional exploration, she begins to identify deeper emotions underlying her anger toward others. For example, when her father discusses her shortcomings in front of outsiders, while the immediate emotion is anger at her father for not understanding her; however, deeper feelings include shame about not being a good daughter, and a profound sense of loneliness and despair caused by his father’s long-term misunderstanding from her father. The therapist works with the patient to deeply explore and experience these emotions, helping her recognize and understand his or her own emotional coping patterns. The therapist also guides the patient in examining specific life events that trigger emotional responses and in identifying multiple emotional layers. For instance, when the patient experiences rejection by peers, she may feel sadness and to fully explore the different levels of emotions the patient is experiencing. For example, when visitors are rejected by students, there will be sad disappointment, followed by anger, there will be deeper self-blame, loneliness, and even despair. Through continuous unconditional acceptance, positive regard, and clarification, the therapist reduces the patient’s fear of emotions, increases the patient’s self-acceptance, and alleviates feelings of loneliness and helplessness.

#### Stage 3: Establishing adaptive emotional processing mechanisms

During the course of therapy, the patient gradually accumulates the positive emotional experiences and emotional processing experiences, leading to the development of a sense of emotional control and the spontaneous transformation of negative emotions. The counselor focuses on and responds to emerging positive emotional expressions, such as “It was not that bad,” helping the patient recognize that he or she is the agent of her emotional experiences. When the patient encounters difficulties in emotional transformation—such as falling into self-denial, avoidance, or unresolved past experiences—the counselor intervenes using repeated chair dialogue techniques. For example, when the patient becomes caught up in the self-critical thoughts such as “I am terrible; I cannot do anything right,” chair dialogue is used to help her express the pain caused by these negative internal voices, articulate her sense of grievance, and identify her genuine needs, thereby strengthening self-protective emotional responses. The empty chair technique is also used to help the patient express repressed emotions related to her relationship with her father. She is encouraged to fully express her anger, dissatisfaction, and unmet needs. She was also encouraged to sit in her father’s seat and to respond from her father’s perspective by switching chairs. After repeated practice, the patient expresses forgiveness toward her mother and mourns her past experiences.

## Results

### Evaluation of the effect of consultation

(1) Self-evaluation of the patient:

The patient’s anxiety and depression symptoms were significantly improved. She was able to communicate with her father at home, and when his speech affected her mood, she could detect these changes, regulate her emotions, and express her feelings. In the classroom can also actively participate in class activities, focus on learning, and make friends. In daily life, physical symptoms (e.g., physical relaxation and relaxed breathing), mood (stability and self-regulation), and behavior (breaking silence and openness to new experiences) all improved after counseling.

(2) Ratings from family and classmates:

The patient was described as less sensitive, more expressive, and occasionally joking.

(3) Psychological assessment—SCL-90-R, SAS, SDS, and TAS-20:

The patient demonstrated good compliance throughout the treatment period and completed the entire course of therapy. No adverse events occurred during this time.

Psychological test results: Standardized assessments confirmed a clinically meaningful improvement in the patient’s psychological functioning. Initially, the patient exhibited scores in the clinical range (SCL-90-R = 190, SDS = 72, SAS = 60, and TAS-20 = 60). After treatment, the scores (SCL-90-R = 140, SDS = 45, SAS = 38, and TAS-20 = 45) all fell below clinical cut-off values. In addition, the number of positive items on the SCL-90-R also decreased from 57 before treatment to 38 after treatment. This change across all measures indicates a successful transition from clinically significant distress to the non-clinical range. As shown in **Table 2**.

**Table 2:** Results of psychological assessment before and after intervention.

Time point	Scl-90-R	SDS	SAS	TAS-20
Pre-intervention	190	72	60	60
Post-intervention	140	45	38	45

Scl-90, Symptom Checklist 90; SAS, Self-Rating Anxiety; SDS, Self-Rating Depression Scale; TAS-20, Toronto Alexithymia Scale.

(4) Therapist evaluation:

The frequency and duration of the patient’s silence gradually decreased. When asked about her feelings, she shifted from denial and avoidance to emotional expression. For example, at the beginning of counseling, the patient’s typical response to the therapist was silence. However, by the 10th session, she spontaneously cried, expressed her vulnerability, and verbalized her emotions. She became increasingly able to identify her emotions, and when experiencing physical discomfort, she no longer focused solely on bodily sensations but also attended to accompanying emotional changes. At the same time, she was better able to describe her emotional states using language. After the intervention, depressive symptoms of depression improved significantly, social functioning was largely restored, sleep quality was good, and counseling essentially achieved the expected goals. The comprehensive evaluation indicates that the patient’s psychological problems were largely resolved.

### Patient

**Figure 1:**
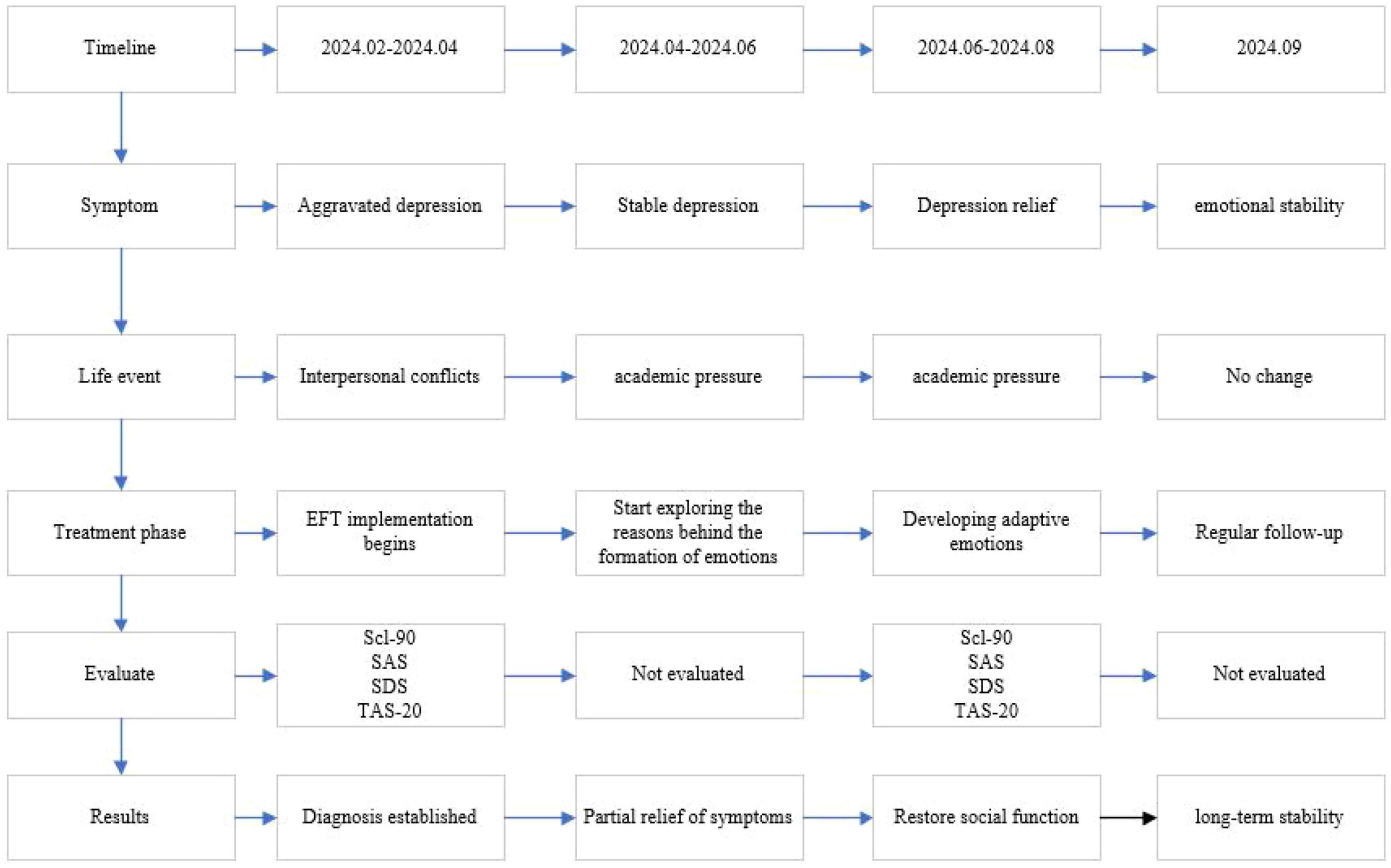
Timeline of patient treatment process.

Self-Report: At the beginning of treatment, the patient could only describe the events occurring around her. When attempting to express emotions, she was often unable to speak and remained unclear or silent. By the eighth session, the patient reported that she gradually began to perceive her emotions in certain situations. For example, she stated, “In the square, when someone greets me, I feel nervous and experience rapid breathing, which makes me feel anxious. “At the 12th session, the patient stated, “I can adjust based on the emotions I feel. I understand the meaning behind emotions, and I try to regulate my emotions myself. It does not last as long as before. When emotions arise, I try to adjust myself.” She also reported that she actively participating in interpersonal communication and conflict resolution, such as talking with friends after disagreements by expressing emotions and feelings and seeking solutions, rather than withdrawing. See **Figure 1**.

## Discussion

This case study explored the effects of emotion-focused therapy (EFT) in a depressed patient with alexithymia. The results show that this intervention can significantly reduce the severity of depression from multiple perspectives. After 18 sessions of treatment, the patient’s negative emotions have been improved, alexithymia was alleviated, and social functioning was restored. EFT demonstrated a positive therapeutic effect for patients with alexithymia and may be considered for broader clinical application.

EFT’s conceptualization of depressive states was appropriately applied in this case. The patient’s depressive state was primarily associated with maladaptive emotional processing mechanisms. Because her childhood needs for love and protection were unmet and her authentic emotional experiences were not recognized, she developed persistent feelings of loneliness, helplessness, and reduced self-efficacy. To cope with this sense of being unprotected state, she began to deny and ignore negative emotions from an early age. By portraying herself as strong and unaffected by negative emotions, she avoided confronting her own helplessness and loneliness. Suppression of her genuine feelings did not reduce their impact; instead, it impaired her ability to process her emotions and seek support, thereby exacerbating loneliness and diminished self-efficacy. Lacking the positive experiences of accepting and managing negative emotions—which she perceived as shameful—she became increasingly self-critical and unable to cope independently.

Emotion-focused therapy is a profound transformative process aimed at reconstructing an individual’s relationship with internal emotional experiences. Its mechanisms of action can be understood across several levels:

(1) EFT establishes a safe therapeutic space through a relationship characterized by deep empathy and acceptance. Within this space, the therapist uses empathic attunement and experiential focusing to guide the client in redirecting attention resources away from excessive focus on external events (externally oriented thinking) toward internal somatosensory and interoceptive signals. This process directly targets the “emotional recognition deficits characteristic of alexithymia. Through systematic and guided exposure to interoceptive sensations, EFT reduces fear and avoidance of emotional experiences, thereby enhancing emotional awareness.

(2) Through guided attention and experiential questioning, the therapist assists the client with localization and embodiment of emotional experience—identifying vague discomfort in specific bodily regions and describing its sensory qualities (e.g., “tightness,” “burning,” and “emptiness”). With the support of emotional vocabulary provided by the therapist, they make preliminary connections are formed between these bodily sensations and specific basic emotions (e.g., sadness, anger, fear, and shame). This process supports the construction of missing “emotional concepts and directly addresses “emotional description deficits, enabling emotional experiences to enter conscious awareness and undergo meaning-making.

(3) Through empathic validation and emotional regulation techniques, the therapist helps the client remain within the “window of tolerance” for emotional work, preventing emotional overwhelm. Building on this foundation, EFT’s core lies in transforming maladaptive emotional schemas. These schemas typically involve inadequately processed core emotions linked to early attachment trauma. Through tasks such as empty chair dialogues or dual-chair dialogues, therapists guide clients to access, activate, and fully express these blocked core emotions (e.g., fear of abandonment and unacknowledged anger). Within the therapeutic encounter, new adaptive emotional responses emerge, such as transforming anger over injustice into self-affirmation, or sorrow over loss into a desire for connection. This transformative process repairs rigid emotional memory networks, shifting the client from emotional control to flexible use of emotional information to guide action.

### Narrative reconstruction and self-integration

As emotional recognition and regulation abilities improve, EFT further assists clients in narrative reconstruction. Clients integrate newly acquired emotional understanding into their life stories. Past experiences that were previously inexpressible—manifesting only as physical symptoms or chaotic behaviors—can now be explained through coherent narratives incorporating emotional motivations and inner needs. For example, “unexplained abdominal pain” may be reframed as “an expression of suppressed anger in stressful situations.” This process enhances self-coherence and agency, marking a key milestone in recovery from affective disorders (21, 22). Although we only report TAS-20 total scores are reported in **Table 2**, inspection of the subscales indicated reductions across difficulty identifying feelings (DIF), difficulty describing feelings (DDF), and externally oriented thinking (EOT), consistent with the focus of EFT on improving emotional recognition, emotional language, and shifting attention from externally oriented thinking toward internal emotional awareness. The observed reductions across all TAS-20 subscales (DIF, DDF, and EOT), although only total scores are presented in **Table 2**, further corroborate the EFT treatment model. This pattern of improvement mirrors the therapeutic focus on fostering emotional identification, cultivating expressive capacity, and reorienting attention from external events to internal emotional experience.

A study of 116 patients with major depressive disorder and 540 control subjects explored the relationship between individual alexithymia and depression. Alexithymia was assessed using the TAS-20, and depression was evaluated using the Beck Depression Inventory (BDI). The results showed that the severity of depression severity was significantly correlated with alexithymia. In addition, BDI scores increased or decreased proportionally with changes in TAS-20 scores in both groups (23, 24). This study emphasizes the role of emotional perception, emotional expression, and emotional experience, as well as the avoidance processes triggered by anticipated fear. The observation results include changes associated with accessing core emotions such as shame, fear, and loneliness, as well as the transformation of changing these emotions through the development of self-compassion and assertive anger (25). The clinical application of EFT warrants further promotion; however, the optimal treatment duration requires additional clarification to support more efficient application of clinical implementation in the future.

### Limitations and future research directions

This study also has several limitations. First, it involved only a single case, and although there is evidence supporting the treatment effect, empirical research in larger populations is still needed. Second, the measurement tools used in this study were self-report scales, which may lack objectivity. Third, this study cannot exclude expectancy effects. Fourth, the study did not employ a double-blind design. Future studies should attempt to examine the therapeutic effects of this counseling approach in large, cross-cultural randomised controlled trials. In addition, given the complexity of patient profiles, future research may explore combining EFT with additional techniques and methods. By integrating the strengths of multiple approaches, it is anticipated that more comprehensive and effective counseling strategies may be developed for individuals who are struggling with depression. Furthermore, combining cognitive neuroscience methods to explore the neural mechanisms underlying treatment effects may provide valuable insights for future research.

### Patient perspective

Throughout treatment, the patient progressed from primarily describing external events and struggling to articulate emotions to identifying feelings in specific situations—for example, recognizing anxiety through physical signs such as rapid breathing. She learned to understand her emotions, apply self-regulation strategies, and reduce the duration of distressing emotional episodes. Notably, she began to actively engage in interpersonal communication and conflict resolution rather than avoidance. This progress fostered a sustained sense of improvement and renewed hope for the future.

## Conclusion

In this case, EFT techniques effectively alleviated the patient’s depressive symptoms. By focusing on bodily sensations and employing exploratory techniques, the patient overcame her avoidance and denial of emotions and gradually connected with her authentic emotional experiences. Reflection on emotional cues clarified her inner needs and enhanced self-understanding. The empty-chair technique was effective in addressing unresolved emotional issues. Nevertheless, future research should further explore the efficacy of EFT and its mechanisms of action across different populations.

## Data Availability

The original contributions presented in the study are included in the article/supplementary material. Further inquiries can be directed to the corresponding author.
